# *In-silico* molecular interactions among the secondary metabolites of *Caulerpa* spp. and colorectal cancer targets

**DOI:** 10.3389/fchem.2022.1046313

**Published:** 2022-12-06

**Authors:** Nazli Mert-Ozupek, Gizem Calibasi-Kocal, Nur Olgun, Yasemin Basbinar, Levent Cavas, Hulya Ellidokuz

**Affiliations:** ^1^ Department of Basic Oncology, Institute of Health Sciences, Dokuz Eylül University, İzmir, Türkiye; ^2^ Department of Translational Oncology, Institute of Oncology, Dokuz Eylül University, İzmir, Türkiye; ^3^ Department of Pediatric Oncology, Institute of Oncology, Dokuz Eylül University, İzmir, Türkiye; ^4^ Department of Chemistry, Faculty of Sciences, Dokuz Eylül University, İzmir, Türkiye; ^5^ Department of Preventive Oncology, Institute of Oncology, Dokuz Eylül University, İzmir, Türkiye

**Keywords:** *Caulerpa*, colorectal cancer, *in silico*, pentose phosphate pathway, oncoinformatics

## Abstract

*Caulerpa* spp. secrete more than thirty different bioactive chemicals which have already been used in cancer treatment research since they play a pivotal role in cancer metabolism. Colorectal cancer is one of the most common cancer types, thus using novel and effective chemicals for colorectal cancer treatment is crucial. In the cheminformatics pipeline of this study, ADME-Tox and drug-likeness tests were performed for filtering the secondary metabolites of *Caulerpa* spp. The ligands which were selected from the ADME test were used for *in silico* molecular docking studies against the enzymes of the oxidative branch of the pentose phosphate pathway (glucose-6-phosphate dehydrogenase and 6-phosphoglutarate dehydrogenase), which is of great importance for colorectal cancer, by using AutoDock Vina. Pharmacophore modeling was carried out to align the molecules. Molecular dynamic simulations were performed for each target to validate the molecular docking studies and binding free energies were calculated. According to the ADME test results, 13 different secondary metabolites were selected as potential ligands. Molecular docking studies revealed that vina scores of caulerpin and monomethyl caulerpinate for G6PDH were found as −10.6 kcal mol-1, −10.5 kcal mol-1, respectively. Also, the vina score of caulersin for 6PGD was found as −10.7 kcal mol-1. The highest and the lowest binding free energies were calculated for monomethyl caulerpinate and caulersin, respectively. This *in silico* study showed that caulerpin, monomethyl caulerpinate, and caulersin could be evaluated as promising marine phytochemicals against pentose phosphate pathway enzymes and further studies are recommended to investigate the detailed activity of these secondary metabolites on these targets.

## 1 Introduction

*Caulerpa*, a green siphonous macroalgae, belongs to the Caulerpaceae family with 97 species. It was first described by J V Lamourox in 1809 and derived from the Greek words caulos (stalk/stem) and erpos (creep) (Mehra et al., 2019). Especially, *C. taxifolia* and *C. cylindracea* (previously known as *C. racemosa* var. *cylindracea*) have attracted attention for the last 30 years due to their invasive properties (Montefalcone et al., 2015) and, more importantly, the various properties of bioactive (especially secondary metabolites) chemicals for defense, communication, growth and development regulation, reproduction, competition, and infection (Erb and Kliebenstein, 2020; Ramawat and Goyal, 2020). *Caulerpa* secrete linear or monocyclic terpenoids that have aldehyde and enol-acetate functional groups (Mehra et al., 2019). The structure of caulerpin (C_24_H_18_N_2_O_4_), a secondary metabolite and pigment of the *C. cylindracea* species, was first described by Aguilar-Santos in 1970 (Aguilar-Santos, 1970). Its molecular weight is 398.418 g/mol and its characterized structure is ‘dimethyl-6,13-dihydrodibenzo [b,i] phenazin-5,12-dicarboxylate methyl ester. Caulersin (C_21_H_14_N_2_O_3_) is another bis-indole alkaloid which is isolated from *C. serrulata* (Su et al., 1997) and from *C. racemosa* (Yang et al., 2014). Its isomers are caulersin A, B, and C. The molecular weight of caulersin is 342.1004 g/mol (Su et al., 1997). It is characterized by its *“central troponoid bridging”* bisindole structure (Su et al., 1997). Caulerpenyne is a sesquiterpenoid-structured secondary metabolite which has some bioactivities such as antiproliferative and apoptotic activities (Cavas et al., 2006) and inhibitors of lipoxygenase (Cengiz et al., 2011) and 5-lipoxygenase (Richter et al., 2014), *etc.* Secondary metabolites of genus *Caulerpa* are responsible for complex modulation network induced in AMPK, ER Stress, mitochondrial stress, PTP1B inhibition and cell cycle stop pathways, metabolic reprogramming in cancer cells, apoptosis and cell cycle arrest in cancer metabolism (Mehra et al., 2019).

Colorectal cancer (CRC) is the second and third most common diagnosed cancer type in women and men in the world, respectively (Dekker et al., 2019). According to the World Health Organization data, in 2018, CRC caused 1.80 million new cases and 862,000 deaths all around the world (WHO, 2018). Since CRC is a common and fatal cancer type, using novel and effective chemicals for treatment is essential.

The pentose phosphate pathway (PPP) is the pivotal pathway for ribonucleotide synthesis and is the main source of NADPH (the reduced form of Nicotinamide Adenine Dinucleotide Phosphate) (Stincone et al., 2015), which is of great importance for fatty acid synthesis and reactive oxygen species scavenging. PPP branches from glycolysis, and it plays a key role in cancer cells (Patra and Hay, 2014). The oxidative phase of PPP is initiated with hexokinase, which converts glucose to glucose 6-phosphate (G6P). G6P oxidizes (dehydrogenated) to 6-phosphogluconolactone by the rate-limiting enzyme (glucose 6-phosphate dehydrogenase (G6PDH)) to yield NADPH (by reducing NADP+) (Stincone et al., 2015). The other NADPH source of PPP is the conversion of 6-phosphogluconate into ribose (ribulose) 5-phosphate by 6-phosphogluconate dehydrogenase (6PGD). Eventually, the regeneration of GSH, synthesis of DNA, fatty acids, and sterols is achieved by producing 2 mol of NADPH per mole of G6P entering the oxidative phase of PPP (Patra and Hay, 2014). In many solid tumors, overexpression of PPP (especially the enzyme 6PGD) has already been observed (Patra and Hay, 2014; Jin and Zhou, 2019). Furthermore, targeting oxidative phase of PPP for mutant *KRAS* colorectal carcinomas prevents the recurrence (Gao et al., 2019). Thus, targeting the PPP is a potentially new target for CRC treatment.

*In-silico* computer-aided methods are commonly used to predict and elucidate the molecular-level behavior of a compound (Dege et al., 2022). Molecular docking is a convenient *in-silico* method which can be used to evaluate the binding affinity of the ligand on the receptor and can predict the position of the these molecules (Trott and Olson, 2010; Mert Ozupek and Cavas, 2017). ADME provides information about *in-silico* ADME behavior which is important for medicinal chemistry (Bocci et al., 2017; Dege et al., 2022; Gokce et al., 2022; Pantaleão et al., 2022). Drug-likeness analysis using *in-silico* is of great importance for evaluating the pharmacokinetic features of fast and cheap (Gokce et al., 2022; Hasan et al., 2022).

In this study, the anticancer activity of phytochemicals of *Caulerpa* spp. were tested on potential targets (G6PDH and 6PGD) against CRC by using *in-silico* pharmacokinetic and pharmacodynamic tools. The aim of this study was to investigate the potential *Caulerpa*-based phytochemicals against fundamental targets (G6PDH and 6PGD) for colorectal cancer treatment.

## 2 Materials and methods

### 2.1 Ligand preparation

The secondary metabolites found in *Caulerpa* spp. Were selected from the literature and organism-specific natural product lists of PubChem; Lotus-the natural products occurrence database (https://pubchem.ncbi.nlm.nih.gov). The three-dimensional (3D) structures of chemicals found in *Caulerpa* spp. were extracted from the PubChem Database. The Canonical smiles formats of the secondary metabolites were drawn using ACD/ChemSketch software. To optimize the geometry and minimize the energy for the selected ligands (secondary metabolites), Open Babel (O’Boyle et al., 2011) minimization tool was used. As force field, uff (universal force field) was selected. Conjugate gradients were selected as optimization algorithm, and total number of steps was set as 200.

### 2.2 Protein preparation

The proteins used in the molecular modeling studies were retrieved from the RCSB Protein Data Bank (https://www.rcsb.org/). The crystal structure of receptors against human colorectal cancer G6PDH (PDBID: 6E08; resolution: 1.90 Å) and 6PGD (PDBID: 4GWK; resolution: 1.534 Å) were extracted. The crystal structures were rebuilt and both water and small molecules were removed. To perform energy minimization and geometry optimization, polar hydrogens were added, and non-polar hydrogens were merged into the molecules by using AutoDock Tools-1.5.6. Before the docking studies, Kollman charges were added, and the related receptors were saved in the PDBQT format.

### 2.3 *In silico* analysis of pharmacokinetic ADME, drug-likeness and toxicity test

The drug-likeness of the compounds found in *Caulerpa* spp. was calculated using SwissADME (http://www.swissadme.ch/)(Daina et al., 2017). The molecular structures of compounds were converted into SMILES format. Only the ligands that could Lipinski’s five rule variations (calculated Log P (CLog P) should be less than five, polar surface area, the number of hydrogen bond donors should be less than five, hydrogen acceptors should be less than ten and the molecular weight should be less than 500) with no more than one violation were used for molecular docking experiments. Toxicity Estimation Software Tool (T.E.S.T.) (Martin et al., 2008) and ProTox-II (http://tox.charite.de/protoc_II; Banerjee et al., 2018) server were used to determine the toxicity estimation of secondary metabolites of *Caulerpa* which were selected from ADME results.

### 2.4 Molecular docking studies

To examine the selected ligands on related receptors, molecular docking experiments were carried out with AutoDock Vina. After the minimization process, the grid box resolution was set at 29.7030, 17.7197, and 29.5355 along the x, y, and z points, respectively, for G6PDH (PDBID: 6E08). To define the binding site for conducting the docking for 6PGD (PDBID: 4GWK), grid box resolution was set at 21.6405, 23.5892, and −2.9280 along the x, y, and z points, respectively. The grid dimensions of all receptors were adjusted to 25 × 25×25 for all molecular docking studies. DHEA and 6aminonicotinamide (6ANA) were used as control ligands. The results of the graphical representations were prepared *via* Maestro Schrödinger.

### 2.5 Pharmacophore model generation and pharmacophore screening

The compounds of *Caulerpa* spp. that showed binding affinities less than −10 kcal/mol (threshold value), were analyzed for pharmacophoric features using the PharmaGist web server (Schneidman-Duhovny et al., 2008). For further studies, ZINCPharmer server (Koes and Camacho, 2012) was used to visualize the best pairwise alignment of ligands (compounds from *Caulerpa* spp.) with the pivot molecule (DHEA or 6ANA). Scores were calculated for each pharmacophore feature by PharmaGist server.

### 2.6 Molecular dynamic simulation for docking validation

The molecular docking simulations of caulerpin, monomethyl caulerpinate and caulersin with glucose-6-phosphate dehydrogenase (G6PDH) and 6-phosphogluconate dehydrogenase (6PGD) proteins were performed using a web-based MD simulation package WebGRO for Macromolecular Simulations (https://simlab.uams.edu/) Simlab, the University of Arkansas for Medical Sciences (UAMS), Little Rock, United States provided by GROMACS-2019.2 (Abraham et al., 2015). PRODRG server (Schüttelkopf and Van Aalten, 2004) was used for the generation of the ligand topology files. GROMOS96 43a1 force field was used for the approximation of the protein-ligand (G6PDH-caulerpin; G6PDH-monomethylcaulerpinate; 6PGD-caulersin) interactions. The triclinic box was filled with SPC water and 0.15 M NaCl (counter ions) to neutralize the system for each ligand-protein complex. The equilibration type was NVT/NPT and Parrinello-Danadio-Bussi thermostat and Parrinello-Rahmanbarostat were used to control the temperature (300 K) and the pressure (atmospheric pressure-1 bar). 5,000 steepest descent was used to minimize the energy of the system. Each protein-ligand complex (G6PDH-caulerpin; G6PDH-monomethyl caulerpinate; 6PGD-caulersin) was simulated for 100 ns. H bonds, the Radius of gyration (Rg), Root Mean Square Deviation (RMSD) and SASA were tested to estimate the complex stability.

### 2.7 Calculation of binding free energy by MM/PB(GB)SA

The best docking poses for each ligand (caulerpin, monomethyl caulerpinate, and caulersin) were rescored. In this study, the binding free energy of the ligands was identified to determine the performance of MM/PB(GB)SA by using Amber package (Fast Amber Rescoring for PPI inhibitors-farPPI; http://cadd.zju.edu.cn/farppi; (Wang et al., 2019). The input files were generated using AutoDock Tools. The force field parameter was set as GAFF2 (for ligand) + ff14SB (for a receptor) and the rescoring procedure was set as PB3 (radii = parse, *γ* = 0.00542, *β* = 0.9200). AM1-BCC method was used to calculate the partial charge of the ligands by using antechamber module of Amber.

## 3 Results

### 3.1 Drug-likeness analysis, ADME and toxicity test analysis of ADME properties

Lipophilicity, water solubility, drug-likeness, medicinal chemistry (leadlikeness) values of 36 metabolites from *Caulerpa* spp. Were obtained using the SwissADME server. The results reveal that the logP of 31 compounds were in the range of 0–5, on the other hand, five of the secondary metabolites (transphytol, alpha-tocospiroA, alpha-tocospirone, trifarin and caulerpicin) were not in the range of Lipinski’s Rule of five (LRo5): 2≤logP≤5). According to the rule of 5, the molecular weight should be 200 ≤ MW ≤ 500. The molecular weights of the 32 compounds were in the acceptable range. However, the MW of sulfoquinovosyldiacyl glycerol, amBiosome, alpha-tocoxylenoxy and caulerpicin do not satisfying the Lipinski Ro5. The number of H-bond acceptors (≤10) and donors (≤5) for 34 (except sulfoquinovosyldiacyl glicerol and amBiosome) and 35 (except sulfoquinovosyldiacyl glicerol) compounds falling in acceptable range, respectively. All the compounds (except sulfoquinovosyldiacyl glicerol) were found to be the range of topological polar surface area (TPSA; <140). The minimum and the maximum numbers of rotatable bonds were found to be 0 and 39, respectively (Table 1). Only the chemicals that were acceptable for LRo5 with no violation were selected for the cheminformatic pipeline and further pharmacodynamic studies. Considering all the obtained results, 13 *Caulerpa-*based phytochemicals (caulerpin, caulerpenyne, caulersin, 10,11-epoxycaulerpenyne, flexilin, racemosin C, racemosin B, caulerprenylol B, caulerprenylol A, monomethyl caulerpinate, α-tocospiro A, α-tocospirone and furocaulerpin) were chosen and used in subsequent steps.

**TABLE 1:** List of pharmacokinetic properties of 36 compounds from *Caulerpa* spp.

Properties	Physicochemical properties								Lipophilicity	Water solubility	Pharmacokinetics	Drug-likeness	Medicinal chemistry
**Parameters**	Molecular weight (g/mol)	Number of heavy atoms	Number of aromatic heavy atoms	Number of rotatable bonds	Number of H-bond acceptors	Number of H-bond donors	Molar reflactivity	TPSA (Å)	Log P_0_/w	LogS (ESOL)	GI absorbtion	Lipinski/violation	Synthetic accessibility
**Compound**													
**Caulerpin**	**398.41**	**30**	**22**	**4**	**4**	**2**	**116.54**	**84.18**	**2.98**	**-5.30**	**High**	**Yes/0**	**2.32**
**Caulerpenyne**	**374.43**	**27**	**0**	**10**	**6**	**0**	**103.18**	**78.90**	**3.79**	**-4.19**	**High**	**Yes/0**	**4.69**
**Caulersin**	**342.35**	**26**	**21**	**2**	**3**	**2**	**102.72**	**74.95**	**2.61**	**-4.97**	**High**	**Yes/0**	**2.34**
**10,11-epoxycaulerpenyne**	**390.43**	**28**	**0**	**10**	**7**	**0**	**102.67**	**91.43**	**3.95**	**-3.30**	**High**	**Yes/0**	**5.22**
**Flexilin**	**320.42**	**23**	**0**	**11**	**4**	**0**	**94.12**	**52.60**	**3.69**	**-4.24**	**High**	**Yes/0**	**3.90**
Trans-phytol	296.53	21	0	13	1	1	98.94	20.23	4.71	-5.98	Low	Yes/1	4.30
Alpha tocopherol quinone	446.71	32	0	15	3	1	140.05	54.37	5.83	-7.14	Low	Yes/1	5.74
Taraxerol	426.72	31	0	0	1	1	134.88	20.23	4.77	-8.34	Low	Yes/1	6.04
Beta-sitosterol	414.71	30	0	6	1	1	133.23	20.33	4.79	-7.90	Low	Yes/1	6.30
Palmitic acid	256.42	18	0	14	2	1	80.80	37.30	3.85	-5.02	High	Yes/1	2.31
Sulfoquinovosyldi acylglycerol	834.15	57	0	37	12	4	228.17	197.33	0	-7.12	Low	No/2	9.02
**Racemosin C**	**372.37**	**28**	**18**	**2**	**4**	**3**	**105.83**	**95.18**	**2.15**	**-4.65**	**High**	**Yes/0**	**3.71**
Caulerchlorin	374.82	27	22	2	2	2	110.27	57.88	2.97	-5.83	High	Yes/1	2.26
Racemosin A	345.33	26	12	2	4	2	101.16	92.34	2.97	-5.83	High	Yes/1	2.26
**Racemosin B**	**314.34**	**24**	**20**	**2**	**2**	**2**	**96.45**	**57.88**	**2.74**	**-5.23**	**High**	**Yes/0**	**3.30**
**Caulerprenylol B**	**248.36**	**18**	**6**	**3**	**2**	**2**	**75.08**	**40.46**	**2.93**	**-3.64**	**High**	**Yes/0**	**3.30**
**Caulerprenylol A**	**258.36**	**19**	**6**	**0**	**2**	**2**	**80.88**	**40.46**	**2.91**	**-3.59**	**High**	**Yes/0**	**4.14**
AmBiosome	924.08	65	0	3	18	12	239.06	319.61	3.76	-5.37	Low	No/3	10
**Monomethyl caulerpinate**	**384.38**	**29**	**22**	**3**	**4**	**3**	**112.22**	**95.18**	**2.11**	**-5.09**	**High**	**Yes/0**	**2.21**
4′,5′-dehydrodiodictyonema A	461.68	33	0	18	4	1	142.05	72.47	5.00	-6.81	High	Yes/1	5.28
Racemobutenolid A	308.5	22	0	11	2	0	96.95	26.30	4.63	-5.47	High	Yes/1	4.65
Racemobutenolid B	308.5	22	0	11	2	0	96.95	26.30	4.63	-5.47	High	Yes/1	4.65
(23E)-3β-hydroxy-stigmasta-5,23-dien-28-one	426.67	31	0	5	2	1	133.21	37.30	4.54	-6.58	Low	Yes/1	6.04
(3b,24R)-stigmasta-5,28-diene-3,24-diol	430.66	31	0	6	3	2	130.08	57.53	4.13	-6.01	High	Yes/1	6.05
(3β,24S)-stigmasta-5,28-diene-3,24-diol	430.66	31	0	6	3	2	130.08	57.53	4.04	-6.01	High	Yes/1	6.05
(22E)-3β-hydroxy-cholesta-5,22-dien-24-one	398.62	29	0	4	2	1	123.6	37.30	4.28	-6.12	High	Yes/1	5.82
Fucosterol	410.67	30	0	4	1	1	132.54	20.23	4.28	-6.12	High	Yes/1	5.82
24R,28S-epoxyfucosterol	426.67	31	0	4	2	1	132.02	32.76	4.87	-6.63	Low	Yes/1	6.35
24S,28R-epoxyfucosterol	426.67	31	0	4	2	1	132.02	32.76	4.97	-6.63	Low	Yes/1	6.35
(3β,23E)-stigmasta-5,23-dien-3,28-diol	428.69	31	0	5	2	2	134.18	40.46	4.82	-6.78	High	Yes/1	6.37
α-tocoxylenoxy	552.87	40	12	14	3	1	176.97	38.69	0	-10.13	Low	No/2	6.14
Cacospongionolide C	324.5	23	0	12	3	1	98.11	46.53	4.29	-5.30	High	Yes/1	4.81
**α-tocospiro A**	**462.7**	**33**	**0**	**13**	**4**	**1**	**139.58**	**63.60**	**5.18**	**-6.53**	**Low**	**Yes/0**	**6.88**
**α-tocospirone**	**462.7**	**33**	**0**	**12**	**4**	**1**	**139.58**	**63.60**	**5.45**	**-6.99**	**Low**	**Yes/0**	**6.65**
**Furocaulerpin**	**272.34**	**20**	**5**	**5**	**3**	**0**	**79.86**	**39.44**	**3.79**	**-4.11**	**High**	**Yes/0**	**4.08**
Trifarin	390.56	28	0	15	4	0	118.16	52.60	5.14	-5.87	High	Yes/1	4.46
Caulerpicin	622.10	44	0	39	2	2	203.36	49.33	9.57	-12.63	Low	No/2	5.88

^a^ The bold values indicate the chemicals that fit Lipinski's Rule of 5.

In the cheminformatic pipeline of the study, computational based *in-silico* toxicity was also used. T.E.S.T. tool and ProTox-II servers were used to identify the adverse effects and toxicity of the 13 selected compounds to evaluate several toxicological parameters (acute toxicity, carcinogenicity, cytotoxicity, hepatotoxicity, immunotoxicity, predicted median lethal dose; LD50 and mutagenicity). ProTox-II results revealed that caulerpin, caulerpenyne, caulersin, flexilin, racemosin C, racemosin B and monomethyl caulerpinate belonging to the toxicity class 4, LD50 range from 500 to 1760 mg/kg, these would be harmful in case oral delivery. (Table 2).

**TABLE 2:** List of toxicity properties of selected *Caulerpa*-based phytochemicals.

Endpoint		Caulerpin	Caulerpenyne	Caulersin	10,11-Epoxycaulerpenyne	Flexilin	Racemosin C	Racemosin B	Caulerprenylol B	Caulerprenylol A	Monomethyl caulerpinate	α-tocospiro A	α-tocospirone	Furocaulerpin
Organ toxicity	Hepatotoxicty	IA	IA	IA	IA	IA	IA	IA	IA	IA	IA	IA	IA	IA
Toxicity	Carcinogenicity	IA	IA	IA	IA	IA	IA	IA	IA	IA	IA	IA	IA	IA
	Cytotoxicity	IA	IA	IA	IA	IA	IA	IA	IA	IA	IA	IA	IA	IA
	Immunotoxicity	IA	IA	IA	A	IA	A	A	IA	IA	IA	IA	IA	IA
	LD50 (mg/kg)	1760	500	500	2,000	710	1760	4,425	5,500	4400	1760	300	300	5000
	Mutagenicity	A	IA	A	A	IA	A	IA	IA	IA	IA	IA	IA	IA
	Toxicity class	4	4	4	5	4	4	4	5	5	4	3	3	5
	AR	IA	IA	IA	IA	IA	IA	IA	IA	IA	IA	IA	IA	IA
	AhR	IA	IA	IA	IA	IA	IA	A	IA	IA	IA	IA	IA	IA
	PPARgamma	IA	IA	IA	IA	IA	IA	IA	IA	IA	IA	IA	IA	IA
	P53	IA	IA	IA	IA	IA	IA	IA	IA	IA	IA	IA	IA	IA
	Heat shock protein	IA	IA	IA	IA	IA	IA	IA	IA	IA	IA	IA	IA	IA
Bioconcentration factor	Log10	0.74	N/A	N/A	N/A	0.93	N/A	1.10	2.20	1.81	0.36	1.24	1.55	N/A
*Daphnia magna* toxicity (48 h)	mg/L	0.67	0.16	0.71	4.27 E-02	0.76	2.15	0.78	3.43	5.74	0.66	0.85	0.70	0.19
Developmental toxicity value		0.90	0.68	0.88	0.68	0.68	0.93	0.82	0.79	0.74	0.97	0.77	0.76	0.62
Fathead minnow (LC50 96h)	mg/L	8.61 E-03	0.32	7.15 E-03	0.12	0.30	3.98 E-03	6.69 E-02	13.52	0.53	3.33 E-02	1.20	0.68	0.44
Mutagenicity (AMES)		N/A	-	N/A	+	-	+	+	-	+	N/A	-	-	-
Oral rat LD50)	mg/L	215.30	N/A	759.69	N/A	8193.16	268.66	N/A	764.83	1857.98	288.29	143.00	268.08	N/A

### 3.2 Molecular docking studies

In this study, molecular docking studies were applied for the investigation of anticancer activity of caulerpin, caulerpenyne, 10,11-epoxycaulerpenyne, caulersin, flexilin, racemosin C, racemosin B, caulerprenylol B, caulerprenylol A, monomethyl caulerpinate, α-tocospiro A, α-tocospirone and furocaulerpin. For anticancer studies, G6PDH and 6PGD, which are crucial for CRC, were selected as receptors.

#### 3.2.1 Glucose 6-phosphate dehydrogenase

**FIGURE 1:**
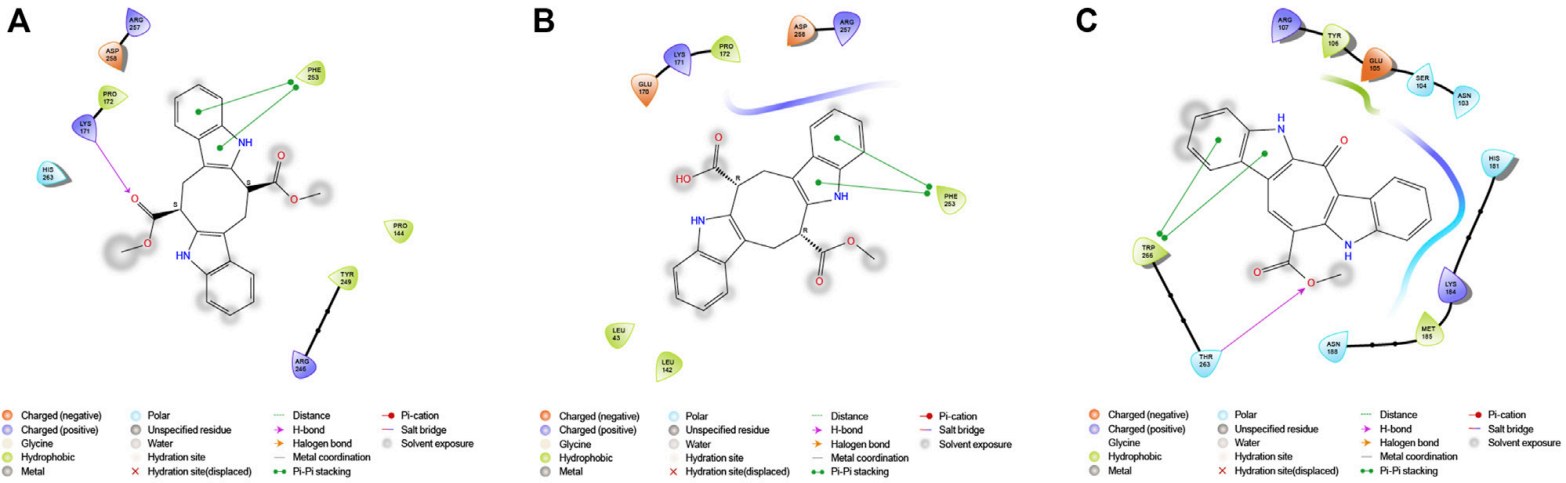
2-D interaction network among **(A)** CPN and amino acid residues of G6PDH, **(B)** MMCPNT and amino acid residues of G6PDH, **(C)** CSN and amino acid residues of 6PGD.

G6PDH is a cytosolic rate-limiting enzyme that converts G6P into 6-phosphoglucono-δ-lactone in the pentose phosphate pathway. In this study, human G6PDH (PDBID:6E08) was selected as a target. Both caulerpin and monomethyl caulerpinate, which have docking scores less than −10 kcal mol^−1^ were selected as ligands with the highest activity. According to the results, the lowest and the highest binding energies on G6PDH were found as −10.6 and −5.8 kcal/mol for caulerpin and 6-aminonicotinamide, respectively (Table 3). Inside the binding cavity of G6PDH, the methyl ester group of caulerpin makes H-bond with Lys171. Also, the indole ring of caulerpin docked in G6PDH makes pi-pi staking with Phe253 (Figure 1A). Furthermore, the indole ring of monomethyl caulerpinate makes pi-pi stacking with Phe253 (Figure1B).

**TABLE 3:** Docking scores with G6PDH and 6PGD and the top 13 selected compounds from *Caulerpa* spp.

Docking score(kcal/mol) compounds		Glucose-6-phosphate dehydrogenase (G6PDH) (PDBID:6E08)	6-Phosphogluconate dehydrogenase (6PGD) (PDBID:4GWK
Caulerpin	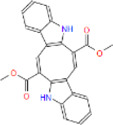	−10.6	−8.7
Caulerpenyne	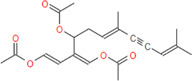	−9.5	−6.8
Caulersin	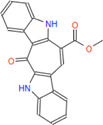	−9.5	−10.7
10,11-epoxycaulerpenyne	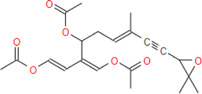	−7.9	−6.5
Flexilin	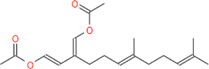	−6.8	−5.9
Racemosin C	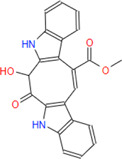	−9.8	−9.1
Racemosin B	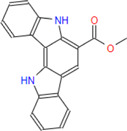	−9.2	−9.5
Caulerprenylol B	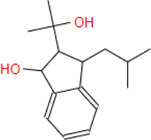	−8.3	−8.3
Caulerprenylol A	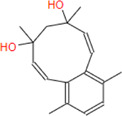	−8.8	−7.7
Monomethyl caulerpinate	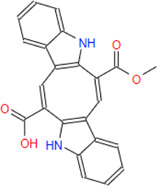	−10.5	−9.8
α-tocospiro A	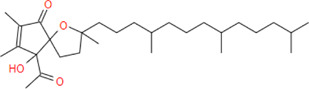	−9.8	−7.3
α-tocospirone	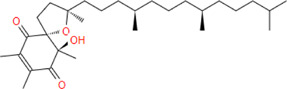	−8.3	−7.4
Furocaulerpin	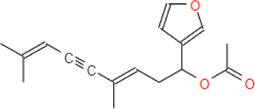	−7.6	−6.9
DHEA		−7.8	−6.2
6 aminonicotinamide		−5.8	−5.8

#### 3.2.2 6-Phosphogluconate dehydrogenase

The molecular docking studies on 6PGD reveal that the lowest binding score was found as −10.7 kcal/mol for caulersin as given in Table 3. Also, the highest binding energy was found as −5.8 for 6-aminonicotinamide. Inside the binding cavity of 6PGD, Trp266 forms pi-pi stacking with both the pirole ring of the indole ring of caulersin. Also, Thr263 makes hydrogen bond with double bond oxygen of the methyl ester group of the central traponoid of caulersin. Related information is given in Figure 1C.

### 3.3 Docking validation by molecular dynamic simulations

**FIGURE 2:**
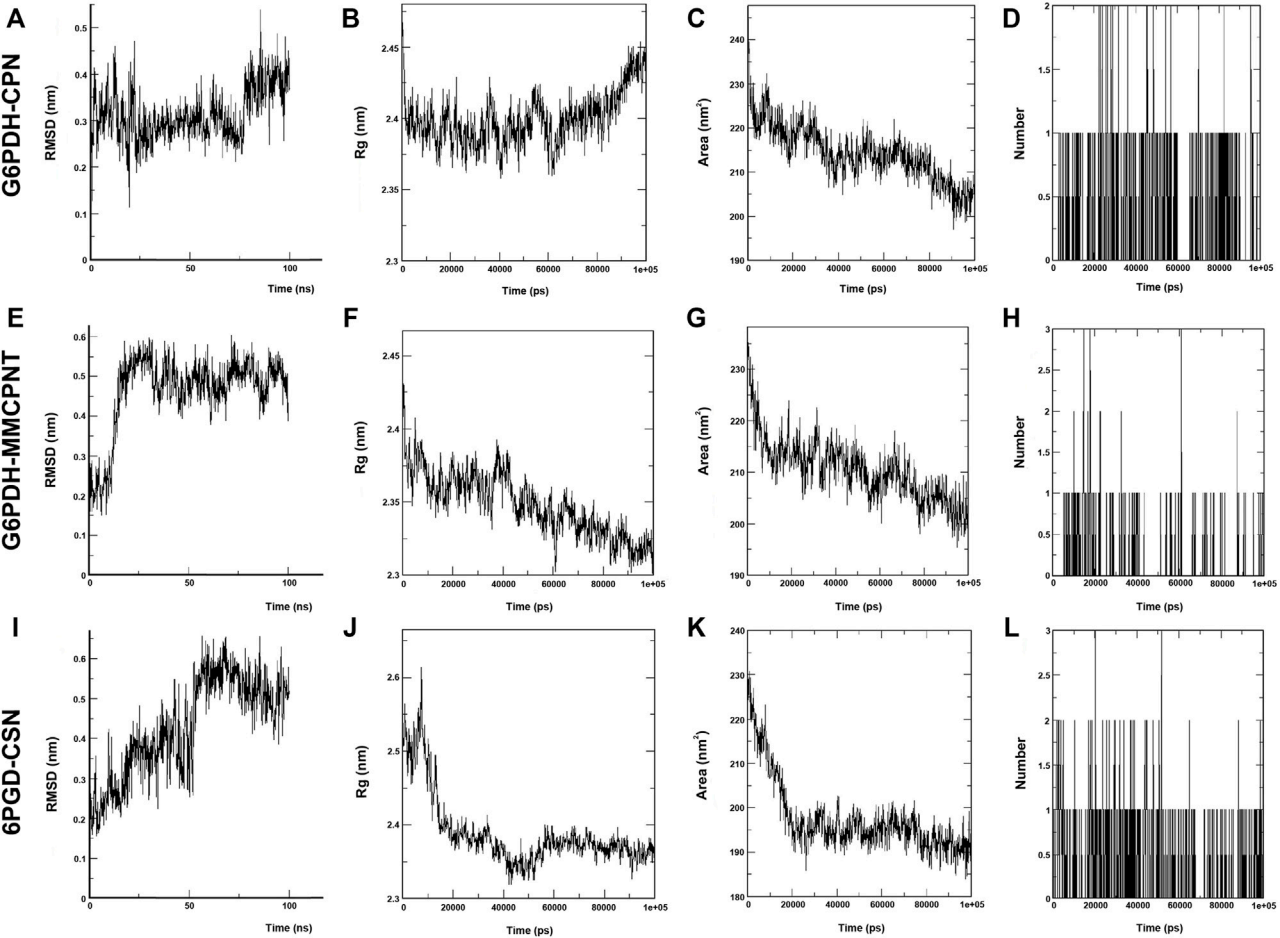
MD simulation of CPN, MMCPNT and CSN with G6PDH and 6PGD; **(A)**, **(E)**, and **(I)** RMSD line plots, **(B)**, **(F)**, and **(J)** Radius of gyration (Rg) line plots, **(C)**, **(G),** and **(K)** SASA line plots, **(D)**, **(H)**, and **(L)**: Line plots of Ligand-protein H bonds for G6PDH-CPN, G6PDH-MMCPNT and 6PGD-CSN, respectively.

In the MD simulations, RMSD (Root Mean Square Deviation) values, the starting position of the backbone of all amino acid residues, were calculated using WebGRO to clarify the stability and overall conformational dynamics of receptor-ligands (G6PDH-caulerpin, G6PDH-monomethyl caulerpinate, 6PGD-caulersin). The results reveal that the average RMSD values for caulerpin-G6PDH, monomethyl caulerpinate-G6PDH and caulersin-6PGD were found as 0.31, 0.46, and 0.43 nm respectively. All the values were comparable and in the physiological environment, indicating the stability of ligand-protein interaction. RMSF (Root Mean Square Fluctuation) values, the standard deviation of atomic positions of each amino acid residues, were also calculated. 0.06, (Figures 2A,E,I). The results from RMSD showed that CPN, MMCPNT and CSN remained positioned at the active sites of the G6PDH and 6PGD with stable interactions.

Radius of gyration (Rg) computes the structural compactness and dynamic adaptability of the ligand-protein complex about the x-, y- and *z*-axes, as a function of time. In Figures 2B,F,J, Rg values of CPN, MMCPNT and CSN with G6PDH and 6PGD receptors ranged between −2.35 and 2.45 nm, −2.30–2.43, and −2.32–2.60 nm, respectively. The overall RG results revealed that, G6PDH-CPN receptor-ligand complex had minimum structural compactness variations and this result indicates the stability of the complexes. SASA is an approximate structural stability of the ligand-protein interaction that is accessible to a solvent (water) with respect to simulation time (100 ns). It was observed that the frequencies of SASA of all G6PDH complexes were decreased around 210 nm^2^ (Figures 2C,G), on the other hand, SASA result of 6PGD-CSN complex was restricted around 190 nm^2^ (Figure 2K). The maximum numbers of H-bonds of caulerpin-G6PDH, monomethyl caulerpinate-G6PDH, and caulersin-6PGD per time frame were found to be 2, 3 and 4, respectively. Furthermore, H-bond formation dynamics between ligands and proteins reveal that for all complexes, at least one H-bond was found as long-lived all through the simulation (100 ns) (Figures 2D, H, L).

### 3.4 Pharmacophore modeling

In this study, combined structure- and ligand-based pharmacophore modeling was performed to evaluate *Caulerpa-*based phytochemicals with potential activity against G6PDH and 6PGD. PharmaGist server was used for pharmacophore modeling to enlighten the three-dimensional pharmacophoric features of top hit ligands for each receptor. Pharmacophore modeling is of great importance for specific receptors to elucidate if the interaction blocks or triggers a biological response.

**FIGURE 3:**
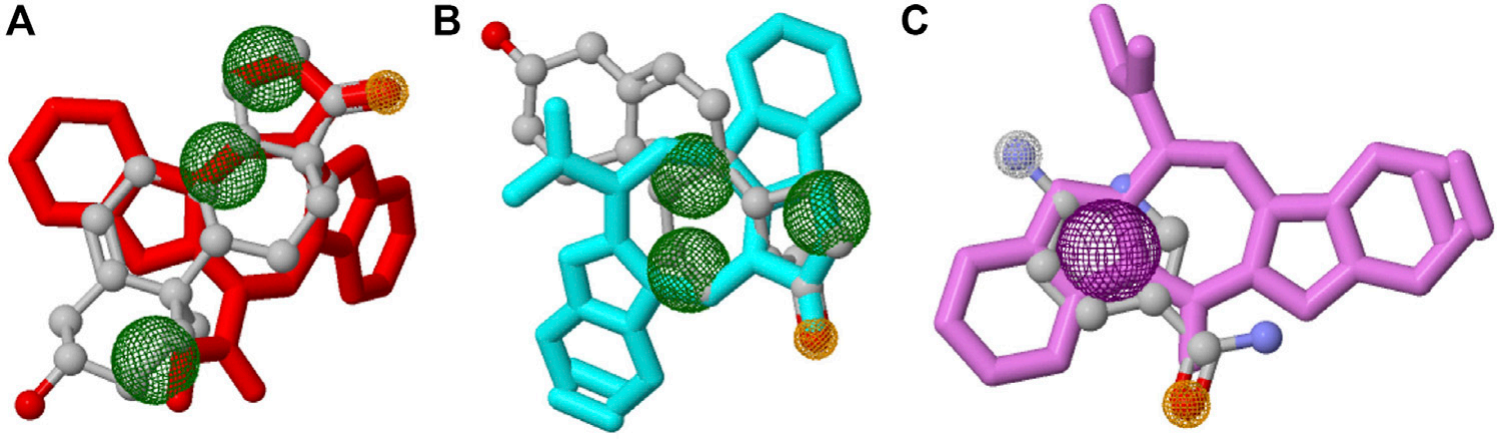
Structural alignment of pivot molecule, **(A)** DHEA (gray) with CPN (red), **(B)** DHEA (gray) with MMCPNT (blue), and **(C)** 6ANA (gray) with CSN (violet). DHEA and 6ANA were displayed in ball and stick style, CPN, MMCPNT and CSN were shown in sticks style. **(A,B)** yellow spheres represent hydrogen bond acceptors, green spheres represent hydrophobic features, **(C)** white spheres represent hydrogen bond donors, yellow spheres represent hydrogen bond acceptors, and purple spheres specify aromatic features.

In this study, top hits (caulerpin and monomethyl caulerpinate for G6PDH and caulersin for 6PGD) were used for each compound in the same orientation at the same binding pocket. The pairwise structural alignment details are given in Table 4. DHEA and 6-aminonicotinamide (6ANA) were used as pivot molecules for G6PDH and 6PGD, respectively. The hit compounds, “caulerpin and monomethyl caulerpinate” and “caulersin” were modeled for G6PDH and 6PGD, respectively. The results revealed that both caulerpin and monomethyl caulerpinate shared the maximum feature number with DHEA. The pairwise structural alignment of DHEA and 6ANA with the top hits is shown in Figure 3.

**TABLE 4:** Pairwise structural alignment showing common pharmacophoric features of secondary metabolites (pivot molecule) and top hit compounds against G6PDH and 6PGD

Score	Features	Spatial features	Aromatic	Hydrophobic	Donors	Acceptors	Negatives	Positives	Molecules
2.430	4	4	0	3	0	1	0	0	DHEA(pivot molecule of G6PDH)-caulerpin (hit compound of G6PDH)
2.431	4	4	0	3	0	1	0	0	DHEA(pivot molecule of G6PDH)-monomethyl caulerpinate (hit compound of G6PDH)
6.016	3	3	1	0	1	1	0	0	6ANA (pivot molecule of 6PGD -caulersin(hit compound of 6PGD)

### 3.5 Binding free energy calculations

**FIGURE 4:**
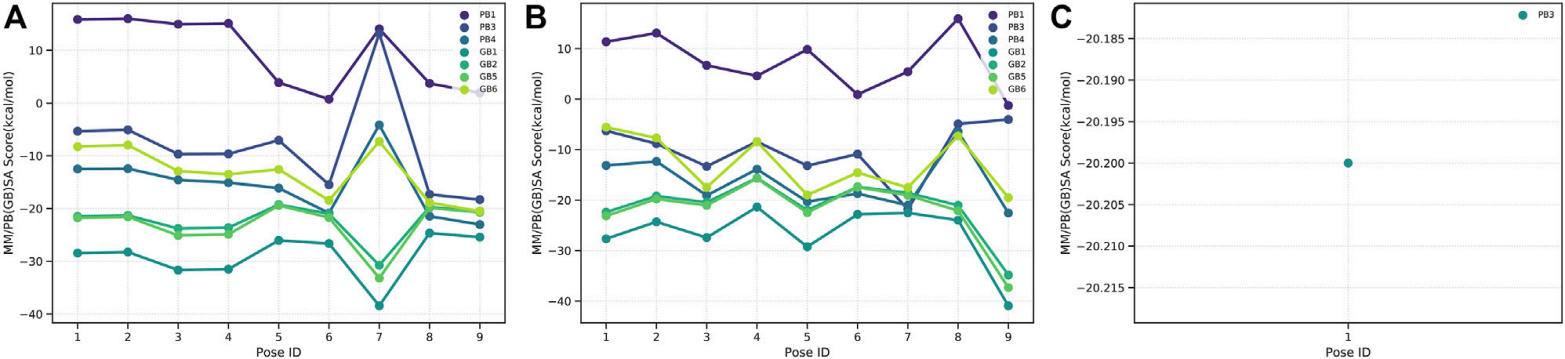
MM-PB(BB)SA results of **(A)** CPN, **(B)** MMCPNT, and **(C)** CSN.

The binding free energies of caulerpin and monomethyl caulerpinate with G6PDH using MM-PB(GB)SA were calculated as -38.43 and -40.94 kcal/mol, respectively (Figures 4A,B). Also, the binding free energy of caulersin with 6PGD using MM-PB (GB) SA was calculated as −20.20 kcal/mol (Figure 4C).

## 4 Discussion

In this study, 13 of 36 different secondary metabolites of *Caulerpa* (caulerpin, caulerpenyne, 10,11-epoxycaulerpenyne, caulersin, flexilin, racemosin C, racemosin B, caulerprenylol B, caulerprenylol A, monomethyl caulerpinate, α-tocospiro A, α-tocospirone and furocaulerpin) against crucial targets (glucose-6-phosphate dehydrogenase and 6-phosphogluconate dehydrogenase) for colorectal cancer were carried out by using *in-silico* pharmacokinetic and pharmacodynamic methods. Caulerpin and monomethyl caulerpinate were found to be the most effective metabolites against G6PDH. Caulersin had the lowest affinity score against 6PGD. The pentose phosphate pathway is fundamental for colorectal cancer, thus caulerpin, monomethyl caulerpinate and caulersin play important roles in colorectal cancer treatment through the pentose phosphate pathway.

ADME is an important medicinal chemistry tool that provides information about *in-silico* ADME behavior (Bocci et al., 2017; Pantaleão et al., 2022). The detailed pharmacokinetic ADME-Tox and drug-likeness results are given in Table 1 and Table 2. The physicochemical properties (molecular weight (g/mol), the number of heavy atoms, the number of aromatic heavy atoms, the number of rotatable bonds, number of H-bond acceptors, the number of H-bond donors, molar refractivity and TPSA (Å)) were calculated for 36 *Caulerpa-*based phytochemicals. ADME results reveal that the compounds with low molecular weight (≤500 g/mol) tend to absorb well (Daina et al., 2017). In our study, 4 of 36 phytochemicals (sulfoquinovosyldiacyl glycerol, amBiosome, alpha-tocoxylenoxy and caulerpicin) from *Caulerpa* spp. have high molecular weight (low absorption capacity). The flexibility of bioactive molecules is determined using the number of rotatable bonds (Daina et al., 2017) which should be between 0 and 9. In this study, all the chemicals except sulfoquinovosyldiacyl glycerol and caulerpicin are in the range of this value. Topological polar surface area (TPSA) is based on the fragmental system of phosphorous atoms and polar sulfur. TPSA value should be between 20 and 130 Å for polarity (Daina et al., 2017). In this study, all the 13 phytochemicals were found in this range. Lipophilicity (logP) is crucial for clarifying the effect of chemicals’ absorption, distribution, transportation on physiological systems. In this study, all the samples (36) out of 5 phytochemicals were in the range of logP (-2≤logP≤5). For water solubility, logS (ESOL) was tested. Daina et al. defined the scale of water solubility as insoluble <−10 < poorly <−6 < moderately <−4 < soluble <−2 < very <0 < highly (Daina et al., 2017). The results reveal that caulerpin, caulerpenyne, caulersin, flexilin, racemosin C, racemosin B, monomethyl caulerpinate, and furocaulerpin were found as moderately soluble; 10,11-epoxycaulerpenyne, caulerprenylol B and caulerprenylol A were found as soluble and α-tocospiro A and α-tocospirone were found as poorly soluble.

Before the clinical trials of drug candidates, *in-silico* toxicity measurement procedure is quite important for better selecting the lead compound (Han et al., 2019). These computational-based toxicity measurement procedures are accurate, accessible, rapid, and common. Both ProTox-II and T.E.S.T servers (freely accessible) were used to identify the adverse effects and toxicity (acute toxicity, carcinogenicity, cytotoxicity, hepatotoxicity, immunotoxicity and mutagenicity) of selected phytochemicals from ADME results. Our results reveal that the toxicity classes of the selected phytochemicals were found to be more than three. For all selected phytochemicals, hepatotoxicity, carcinogenicity, cytotoxicity androgen receptor results were found as inactive.

Drug design and discovery is a step-by-step process, which is costly for companies (Opo et al., 2021). The bioavailability and drug-likeness analysis using *in-silico* is of great importance for evaluating the pharmacokinetic features of fast and cheap (Hasan et al., 2022).

In this study, the selected metabolites (ligands) have the potential anticancer activity against the selected receptor targets for CRC. According to the results, among the commercial drugs (DHEA and 6ANA), for G6PDH, the best docking energy was exhibited by caulerpin with a vina score of −10.6 kcal/mol, while for G6PDH, the other best docking energy was exhibited by monomethyl caulerpinate with a vina score of −10.5 kcal/mol. For 6PGD, the best docking score among the secondary metabolites of *Caulerpa* was exhibited by caulersin (−10.7 kcal/mol). (Table 3). In the literature, limited studies are related to the activities of caulerpin on different targets *in-silico*. In the study by Lorenzo et al. (2015), *in-silico* molecular docking study of caulerpin and its nine analogs against monoamine oxidase B was carried out. Their results reveal that moldock energy, predicted probability (%) and drug-like score of caulerpin were found as −152%, 58%, and 0.77, respectively. However, the analogs of caulerpin which have non-polar and polar groups showed different moldock energy, predicted probability (%) and drug-like scores. The methods that they used (Volsurf descriptors, structure-based methodology and Random Forest algorithm) are crucial for finding the good drug candidates (caulerpin and its analogs) against monoamine oxidase B (Lorenzo et al., 2015). In the literature, Vitale et al. (2018) carried out an *in silico* molecular docking evaluation of caulerpin against PPARalpha and PPAR gamma. Their study reveals that the main interaction between ligand (caulerpin) and protein is a hydrophobic interaction. Also, according to their molecular dynamics results, caulerpin makes intermolecular H-bonds with S289 (VI) and S342/R288 (V) (Vitale et al., 2018). Furthermore, antiviral activity of caulerpin against SARS-CoV-2 was tested using *in-silico* tools (Abdelrheem et al., 2020; Ahmed et al., 2020; Çavaş et al., 2020; El-Mageed et al., 2021).

MM-PBSA analysis is an important and popular method in drug candidate filtration since it is an easy method, and the speed-accuracy balance of the information is high. Estimation of binding free energies with MM-PB(GB) SA for the ligands is automated with the farPPI web server. In our study, the highest and the lowest binding free energies were calculated for monomethyl caulerpinate and caulersin, respectively.

Caulerpin also has some biological activities such as anticancer activity on melanoma cells (Rocha et al., 2007), HIF-1 activation and inhibition of mitochondrial respiration (Liu et al., 2009), antiviral activities against bovine viral diarrhea virus in cattle and herpes simplex virus (Macedo et al., 2012; Pinto et al., 2012; Zhang et al., 2015), pain-sensitizing and spasmolytic effect (Cavalcante-Silva et al., 2014), antituberculosis activity (Canché Chay et al., 2014), antiproliferative activity (Movahhedin et al., 2014), monoamine oxidase inhibitory activity against Alzheimer’s and Parkinson’s disease (Lorenzo et al., 2015), activity on cisplatin-resistant overian cancer and inhibition respiratory complex II activity (Ferramosca et al., 2016), AMPKα1 pathway activation in colorectal cancer cells (Yu et al., 2017) and PPARα and PPARγ agonist activity on hepatocellular cell line (Vitale et al., 2018). Caulersin is a known human protein tyrosine phosphatase-1B inhibitor, which regulates insulin signaling negatively (Yang et al., 2014). The anticancer activity of caulerpin, caulersin, caulerpenyne and 10,11-epoxycaulerpenyne for colorectal cancer data reveal that caulerpin and caulersin are promising anticancer agents against CRC targets and G6PDH and 6PGD could be important targets for CRC.

Inhibition of PPP-enzymes is related with AMPK-activation, HIF-1α degradation, impaired folate metabolism and PP2A-activation (Meskers et al., 2022). G6PDH is the main NADPH production and redox homeostasis contributor (Ghergurovich et al., 2020). The expression level of G6PDH is upregulated and negatively correlated with patients with cancer (Ghergurovich et al., 2020). In different CRC cell lines, the expression levels of G6PDH and 6PGD are quite different. In the study of Polat et al. (2021), the highest and the lowest G6PDH levels were found in HT29 and Caco-2 cell lines, respectively (Polat et al., 2021). Thus, the effectiveness of the selected secondary metabolites could be changed depending on the colorectal cancer cell type.

For the prospects, the not only the *in-silico* analysis but also the *in-vitro* experiments of these targets for caulerpin, monomethyl caulerpinate and caulersin should be performed.

## 5 Conclusion

Global warming and human activities change the ecosystem structures. Alien members of Genus *Caulerpa* are widely studied marine algae due to their invasive properties. The present paper proposes an alternative utilization method in medicinal chemistry. The secondary metabolites of *Caulerpa s*pp. attract attention due to their bioactivities. Using the ADME-tox and drug-likeness tests, 13 of 36 secondary metabolites were selected and molecular docking, and molecular dynamics analysis were performed. Caulerpin, monomethyl caulerpinate, and caulersin were found the hit compounds of *Caulerpa* spp. Against G6PDH and 6PGD, which may play pivotal roles in CRC. Thus, instead of eradication of these algae, the secondary metabolites proposed in this paper (caulerpin, monomethyl caulerpinate) might further be evaluated as promising agents that can be obtained from pharmacy of nature.

## Data Availability

The datasets generated and/or analysed during the current study are available from the corresponding author on reasonable request.
